# Construction and validation of log odds of positive lymph nodes (LODDS)-based nomograms for predicting overall survival and cancer-specific survival in ovarian clear cell carcinoma patients

**DOI:** 10.3389/fonc.2024.1370272

**Published:** 2024-03-21

**Authors:** Zesi Liu, Chunli Jing, Yashi Manisha Hooblal, Hongxia Yang, Ziyu Chen, Fandou Kong

**Affiliations:** ^1^ Department of Gynecology and Obstetrics, The First Affiliated Hospital of Dalian Medical University, Dalian, Liaoning, China; ^2^ Department of Gynecology and Obstetrics, The Second Affiliated Hospital of Dalian Medical University, Dalian, Liaoning, China

**Keywords:** LODDS, ovarian clear cell carcinoma, nomogram, overall survival, cancerspecific survival

## Abstract

**Background:**

Ovarian clear cell carcinoma (OCCC) is one of the special histologic subtypes of ovarian cancer. This study aimed to construct and validate log odds of positive lymph nodes (LODDS)-based nomograms for predicting the overall survival (OS) and cancer-specific survival (CSS) in patients with OCCC.

**Methods:**

Patients who underwent surgical treatment between 2010 and 2016 were extracted from the Surveillance Epidemiology and End Results (SEER) database and the data of OCCC patients from the First Affiliated Hospital of Dalian Medical University were used as the external validation group to test the validity of the prognostic model. The best-fitting models were selected by stepwise Cox regression analysis. Survival probability was calculated by the Kaplan–Meier method, and the differences in survival time between subgroups were compared using the log-rank test. Each nomogram’s performance was assessed by the calibration plots, decision curve analysis (DCA), and receiver operating characteristics (ROC) curves.

**Results:**

T stage, distant metastasis, marital status, and LODDS were identified as significant risk factors for OS. A model with four risk factors (age, T stage, stage, and LODDS value) was obtained for CSS. Nomograms were constructed by incorporating the prognostic factors to predict 1-, 3- and 5-year OS and CSS for OCCC patients, respectively. The area under the curve (AUC) range of our nomogram model for OS and CSS prediction ranged from 0.738-0.771 and 0.769-0.794, respectively, in the training cohort. The performance of this model was verified in the internal and external validation cohorts. Calibration plots illustrated nomograms have good prognostic reliability.

**Conclusion:**

Predictive nomograms were constructed and validated to evaluate the OS and CSS of OCCC patients. These nomograms may provide valuable prognostic information and guide postoperative personalized care in OCCC.

## 1 Introduction

Ovarian cancer is one of the most common malignancies of the female reproductive tract, of which 90% are epithelial ovarian cancer (EOC) (1). Approximately 230,000 people are diagnosed with EOC each year, resulting in 150,000 deaths annually (2).Ovarian clear cell carcinoma (OCCC) is one of the special histologic subtypes of EOC, accounting for about 5% of EOC in western countries, and approximately 20% in Asian countries (3). Compared with EOC, OCCC is more refractory to platinum-based first-line chemotherapy, with the response rate in OCCC being 11.56% (4, 5). Although early-stage OCCC has a relatively good prognosis, with a 5-year survival rate of 90%, the median overall survival time in advanced-stage OCCC is significantly shorter than that in high-grade serous ovarian cancer (HGSOC) (6, 7). Lymph node (LN) metastasis is one of the main metastasis modes of OCCC (8). The status of regional lymph nodes (LNs) retrieved during surgery appears to be not only an independent prognostic factor but also an essential factor in assessing the risk of recurrence of patients with OCCC (9). The American Joint Committee on Cancer/International Union Against Cancer (AJCC/UICC) tumor-node-metastasis (TNM) classification is widely used to predict prognosis but may lead to an underestimation of N-stage due to its calculation only based on the absolute number of positive LNs. Therefore, many novel LNs staging systems have been proposed to improve the assessment of prognosis in OCCC.

Log odds of positive lymph nodes (LODDS) comprehensively considers the effect of the number of positive lymph nodes (PLNs) and resected lymph nodes (RLNs) on the prognosis for tumor patients and has been widely proven as an effective prognosis prediction tool and a novel lymph node staging system in various malignancies (10). LODDS is calculated with the following expression:


Log [(PLNs+0.5)/(RNs−PLNs+0.5)]

In addition, compared with the AJCC N stage, LODDS showed better discrimination abilities and well-fitting in predicting survival in patients with stage IV rectal cancer (11).

Based on entropy, the Akaike Information Criterion (AIC) statistic calculates the tradeoff between overfitting and poor-fitting models and takes into account the number of parameters that the model estimates to select the more parsimonious model (12, 13). The corrected Akaike Information Criterion (AICc) is a modified version of the AIC including a correction term for small sample sizes and is calculated as following:


AICc= AIC+[2k(k+1)]/(n−k−1)

The k denotes the number of free parameters, and n is the number of observations (14, 15). In this study, we aimed to use AICc to build prognostic models of the overall survival (OS) and cancer-specific survival (CSS) for OCCC. Finally, nomogram is used to integrate multiple prognostic factors, which enables it to predict a patient’s survival with relative accuracy (16).

## 2 Materials and methods

### 2.1 Data source and study population

The Surveillance, Epidemiology, and End Results (SEER) database is supported by the national cancer institute (NCI) of USA and has been around since 1973. The SEER database collects information on every case of cancer reported in 19 geographic regions of the U.S., accounting for about 34.6% of the U.S. population. The SEER∗Stat software (version 8.3.6, https://seer.cancer.gov/seerstat/) was used to screen eligible patients who were OCCC between 2010 and 2016. According to the International classification of Diseases for Oncology, 3rd edition (ICD-O-3) morphological code, histopathologic classification of patients was performed, and the subtypes included: 8310/3, 8313/3, 8443/3 and 8444/3. At the same time, in order to increase the reliability of the results of this trial and to minimize experimental bias, data of OCCC patients from the Department of Gynecology of the First Affiliated Hospital of Dalian Medical University from June 2011 to June 2021 were used as the external validation group to test the validity of the prognostic model (n = 50).

Exclusion criteria are as follows: (a) No histologic diagnosis; (b) Contain two or more primary malignancies; (c) Survival months less than one month; (d) Treatment by primary site surgery; (e) ≥18 years of age; (f) Complete LN data; (g) Lack of relevant demographic and clinicopathological characteristics.

### 2.2 Variables collected

The following variables for this study were extracted: age, race, marital status, grade (G1 is equivalent to well differentiated; G2 is equivalent to moderately differentiated; G3 is equivalent to poorly differentiated; G4 is equivalent to undifferentiated), 7th AJCC stage, 7th AJCC TNM stage, tumor size, chemotherapy record, RLNs, PLNs, organ metastasis. OS and CSS were considered the primary endpoints. The cut-off values were established by *X-tile* program (3.5.1) (17).

### 2.3 Statistical analysis

All OCCC patients from the SEER database were assigned as the training group, and 30% of them were selected by random sampling as the internal validation group. All 50 OCCC patients collected from the First Affiliated Hospital of Dalian Medical University were used as external validation group. Baseline differences in demographic variables between the training cohort and validation cohort were investigated using chi-square tests and independent-sample *t* tests. Survival probability was calculated by the Kaplan–Meier method, and the differences in survival time between distinct subgroups were compared using the Log-rank test. To identify significant univariate results, the univariate results were visually inspected in R software by comparing the cumulative incidence function (CIF) based on the Turnbull estimator to the cumulative incidence function based on the normal distribution. The Akaike Information Criterion, corrected for small sample size was determined; a smaller AICc means a better fit, and was penalized for being overloaded with parameters (18, 19). As a result, the best-fitting model was chosen by selecting the lowest *AICc*. Then, nomograms were constructed and used to predict 1-, 3- and 5-year OS and CSS for OCCC patients. The predictive performance of the nomogram was verified internally for discrimination and calibration through the *C*-statistics, area under the curve (AUC) and calibration curves (20, 21). Finally, by evaluating model performance by considering the clinical consequences of true positives and false positives, decision curve analysis (DCA) compares the net benefit between the nomogram model and the multivariate *Cox* regression model across a range of threshold probabilities so that we can select better predictive models for clinical decision making.

All statistical analyses were performed with R version 4.2.1 (www.R-project.org). A *P*-value of< 0.05 was considered statistically significant.

## 3 Results

### 3.1 Patient characteristics

**Figure 1:**
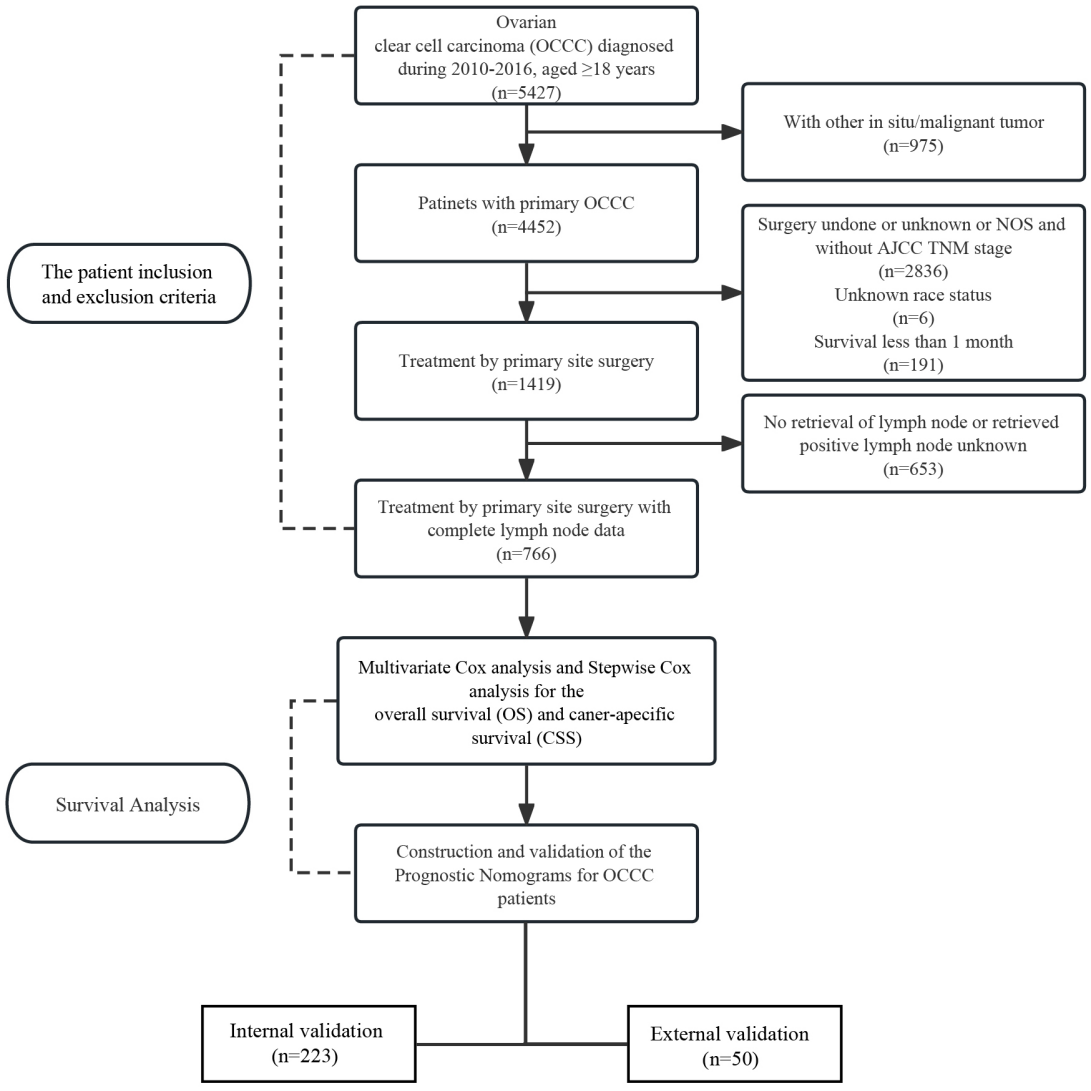
Flow diagram of patient selection and survival analysis.

A total of 766 patients with primary OCCC from the SEER database were enrolled in the trial, and data on 50 patients with primary OCCC were collected as an external validation group for the trial (**Figure 1**) and the characteristics of these patients from the SEER database are listed in ​**Table 1**. There were no significant differences between the training group and the validation group with regards to the demographic and clinicopathological characteristics, thus implying that two groups were comparable. The incidence of OCCC is higher in the elderly, with 86.5% of patients older than 45 years. The distribution of race among patients demonstrated that the largest ethnic groups were white people (72.1%). Although most patients were diagnosed at a limited stage (64.8%), 53.4% had poorly differentiated tumors, 36.6% had undifferentiated tumors and 82.1% received chemotherapy during treatment in the training cohort.

**Table 1:** Patients’ demographics and clinicopathological characteristics.

Characteristic	Training cohort (n=766)	Internal validation group (n=223)	External validation group (n=50)	*P*-value
Age (years), n (%)				
Mean ± SD	55.7 ± 10.2	54.5 ± 9.7	52.9 ± 8.4	0.769
18-44	103 (13.5)	29 (12.9)	10(20.0)	0.842
45-52	197 (25.7)	58 (26.1)	16(32.0)	
>52	466 (60.8)	136 (61.0)	24(48.0)	
Race, n (%)				
White	552 (72.1)	161 (72.4)	–	0.961
Black	27 (3.5)	8 (3.7)	–	
Other/Unknown	187 (24.4)	54 (23.9)	–	
Grade, n (%)				
Well differentiated (G1)	9 (1.2)	2 (0.9)	10(20.0)	0.944
Moderately differentiated (G2)	68 (8.9%)	18 (8.2)	8(16.0)	
Poorly differentiated (G3)	409 (53.4)	121 (54.3)	20(40.0)	
Undifferentiated (G4)	280 (36.6)	82 (36.6)	12(24.0)	
AJCC T Stage, n (%)				
T1	532 (69.5)	154 (69.2)	29(58.0)	0.909
T2	110 (14.4)	31 (13.8)	11(22.0)	
T3	124 (16.2)	38 (17.0)	10(20.0)	
AJCC N Stage, n (%)				
N0	645 (85.4)	191 (85.6)	40(80.0)	0.961
N1	112 (14.6)	32 (14.4)	10(20.0)	
AJCC M Stage, n (%)				
M0	736 (96.1)	213 (95.3)	47(94.0)	0.980
M1	30 (3.9)	10 (4.7)	3(6.0)	
Stage, n (%)				
I	496 (64.8)	145 (64.7)	28(56.0)	0.948
II	89 (11.6)	24 (10.8)	11(22.0)	
III	151 (19.7)	46 (20.7)	8(16.0)	
IV	30 (3.9)	8 (3.7)	3(6.0)	
Chemotherapy, n (%)				
Yes	629 (82.1)	181 (81.2)	42(84.0)	0.713
No	137 (17.9)	42 (18.8)	8(16.0)	
Marital status, n (%)				
Married	428 (55.9)	125 (56.0)	29(58.0)	0.934
Unmarried	338 (44.1)	98 (44.0)	21(42.0)	
Tumor size (mm)				
Mean ± SD	123 ± 4.2	121 ± 3.8	124 ± 4.4	0.936
<85	209 (27.3)	60 (26.7)	14(28.0)	0.728
85-179	411 (53.7)	119 (53.2)	27(54.0)	
≥180	146 (19.0)	44 (20.1)	9(18.0)	
CA125, n (%)				
Negative/Unknown	334 (43.6)	96 (43.1)	12(24.0)	0.871
Positive	432 (56.4)	127(56.9)	38(76.0)	
RLNs (Mean ± SD)	16.3 ± 11.9	16.6 ± 12.4	15.9 ± 12.7	0.646
PLNs (Mean ± SD)	0.56 ± 2.23	0.52 ± 2.10	0.59 ± 2.31	0.733
LODDS (Mean ± SD)	-1.26 ± 0.55)	-1.27 ± 0.54	-1.28 ± 0.52	0.727

AJCC, American Joint Committee on Cancer; RLNs, resected lymph nodes; PLNs, positive lymph nodes; LODDS, Log odds of positive lymph nodes.

^£^: P-value with Bonferroni adjustment.

### 3.2 Survival analysis

**Figure 2:**
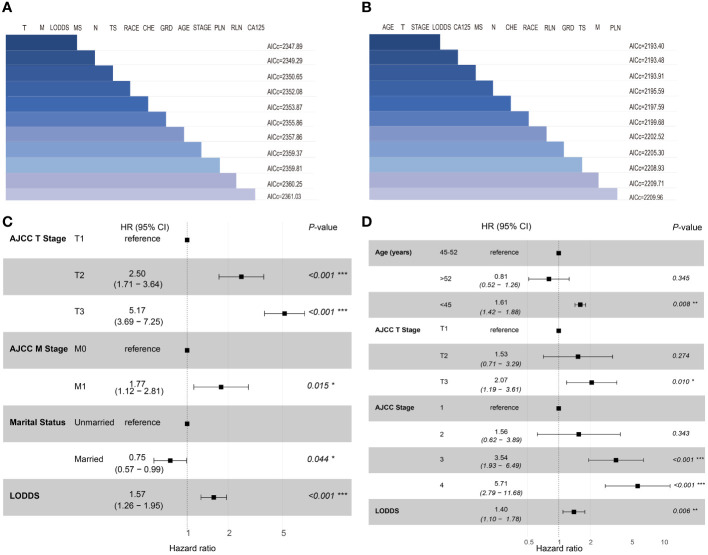
Set of models created with forward-stepwise selection for OS **(A)** and CSS **(B)**, ranked by AICc. Shaded boxes signify the factors included within the model. Forest plots of independent risk factors in stepwise Cox regression analysis of OS **(C)** and CSS **(D)**. T, 7th AJCC T Stage; M, 7th AJCC M Stage; MS, Marital status; N, 7th AJCC N Stage; TS, Tumor size; CHE, Chemotherapy; GRD, grade; PLN, positive lymph node; RLN, resected lymph node.

In this study, the 14 variables included were analyzed by multivariate *Cox* analysis and stepwise *Cox* regression analysis. The results of multivariate *Cox* analysis indicated that Blacks (HR:2.27, 95% CI:1.03-5.00; P=0.042), AJCC stage III (HR:3.23, 95% CI:1.45-7.20; P=0.004), AJCC stage IV (HR:5.08, 95% CI:2.17-11.90; P<0.001), AJCC T3 stage (HR:2.20, 95% CI:1.12-4.30; P=0.022), distant metastasis (HR:1.69, 95% CI:1.12-2.17; P=0.014), and LODDS value (HR:1.61, 95% CI:1.00-2.60; P=0.048) were risk factors of OS. The OS was better for married OCCC patients (HR:0.79, 95% CI:0.57-0.91; P=0.043) (**Supplementary Figure 1**). By comparing the goodness-of-fit AICc statistics of model performance, the model with the lowest AICc value was the best-fitting model (22) (**Figure 2A**). As a result, AJCC T2 stage (HR:2.50, 95% CI:1.71-3.64; P<0.001), AJCC T3 stage (HR:5.17, 95% CI:3.69-7.25; P<0.001), distant metastasis (HR:1.77, 95% CI:1.12-2.81; P=0.015), marital status (HR:0.75, 95% CI:0.57-0.99; P=0.044), and LODDS (HR:1.57, 95% CI:1.26-1.95; P<0.001) were screened and identified as significant risk factors for OS in OCCC patients (**Figure 2C**).

**Figure 3:**
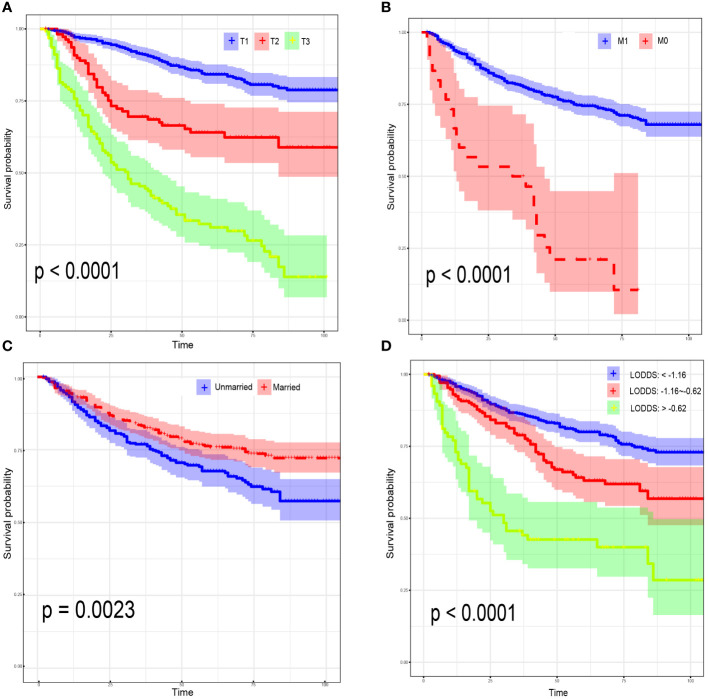
Kaplan–Meier curves for overall survival, stratified by 7th AJCC T Stage **(A)**; 7th AJCC M Stage **(B)**; marital status **(C)**; LODDS **(D)**.

For CSS, age<45 years old (HR:1.64, 95% CI:1.43-1.94; P=0.021), AJCC stage 3 (HR:4.23, 95% CI:2.07-8.63; P<0.001), AJCC stage 4 (HR:6.23, 95% CI:2.80-13.83; P<0.001), distant metastasis (HR:1.91, 95% CI:1.47-2.71; P=0.039) and LODDS value (HR:1.68, 95% CI:1.12-2.51; P=0.012) were identified as risk factors. Interestingly, OCCC patients with evaluative CA125 indicated better CSS (HR:0.66, 95% CI:0.43-1.00; P=0.050) (**Supplementary Figure 2**). Similarly, a model with the lowest AICc value (**Figure 2B**) included four risk factors: age<45 years old (HR:1.61, 95% CI:1.42-1.88; P=0.008), AJCC T3 stage (HR:2.07, 95% CI:1.19-3.61; P=0.010), AJCC stage III (HR:3.54, 95% CI:1.93-6.49; P<0.001), AJCC stage IV (HR:5.71, 95% CI:2.79-11.68; P<0.001) and LODDS value (HR:1.40, 95% CI:1.10-1.78; P=0.006) was screened to predict CSS (**Figure 2D**). The Log-rank test was also used to explore differences in survival between subgroups based on risk factors and these results were visualized using Kaplan–Meier curves. According to the Kaplan-Meier survival curves in **Figures 3A–D**, there were significant differences in survival in AJCC T stage (P<0.001), organ metastasis (P<0.001), marital status (P=0.002), LODDS value (P<0.001) subgroups. In terms of competing risks, CIF curves were implemented to the risk factors according to CIF values for cancer-specific death (**Supplementary Figures 3A–D**).

### 3.3 Construction and validation of the prognostic nomograms

**Figure 4:**
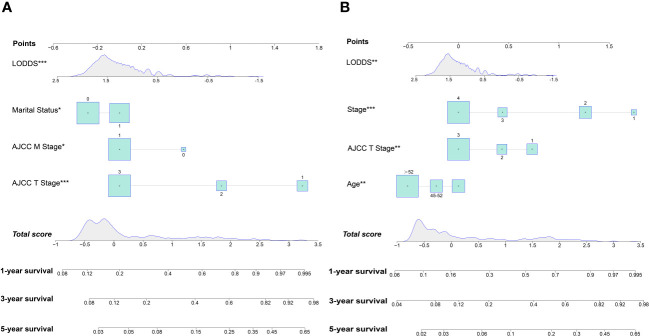
Nomogram for predicting 1-, 3- and 5-year OS **(A)**; Nomogram for predicting 1-, 3- and 5-year CSS **(B)**.

**Figure 5:**
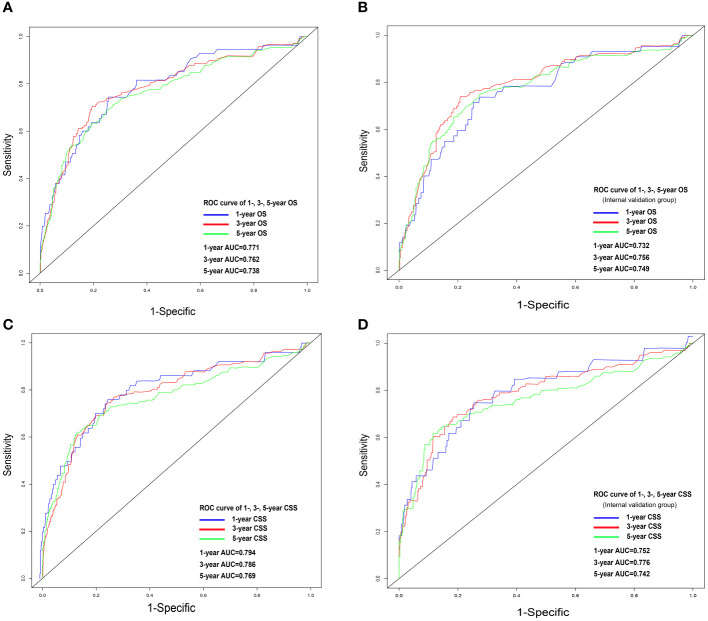
ROC analysis for OS and CSS. OS nomogram ROC curve for training cohort **(A)** and internal validation cohort **(B)**; CSS nomogram ROC curve for training cohort **(C)** and internal validation cohort **(D)**. OS, overall survival; CSS, cancer-specific survival; ROC, receiver operating characteristics.

**Figure 6:**
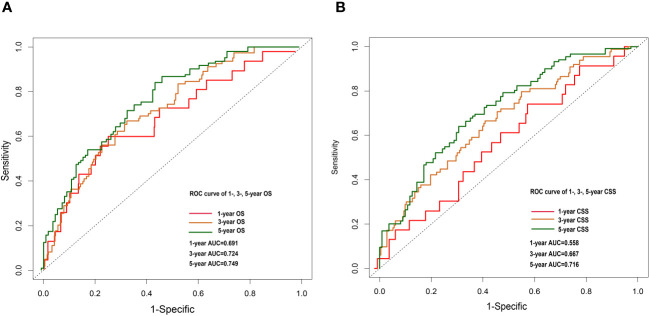
1-, 3- and 5-year OS nomogram ROC curve for external validation cohort **(A)**. 1-, 3-and 5-year CSS nomogram ROC curve for external validation cohort **(B)**.

Nomograms were constructed by incorporating the prognostic factors to predict 1-, 3- and 5-year OS (**Figure 4A**) and CSS (**Figure 4B**) for OCCC patients. The C-statistic ranges from 0.5, which indicates the absence of discrimination, to 1.0, indicating perfect discrimination. Generally speaking, if the C-statistic value is greater than 0.7, the model has very good predictive value (23, 24). The C-statistic values of our nomogram model for OS and CSS prediction were 0.756 (95% CI: 0.728-0.764) and 0.746 (95%CI: 0.744-0.748), which denoted the good performance of the nomogram models. The actual survival rates of OCCC showed a good agreement with the optimal bootstrap predicted values, indicating good prognostic reliability (**Supplementary Figures 4–7**). The AUC values also indicated the nomogram had favorable sensitivity and specificity in predicting OS (**Figures 5A, B**) and CSS (**Figures 5C, D**) in OCCC patients. Additionally, the DCA curve indicated that the nomogram models had better prediction performance than the multivariate *Cox* regression model (**Supplementary Figures 8, 9**). Similar results were observed in the internal validation cohort. Finally, the real-world data was utilized for external validation. The 1, 3, 5-year AUC area was 0.691, 0.724 and 0.749 for OS, and the 1, 3, 5-year AUC area was 0.558, 0.667 and 0.716 for CSS, respectively (**Figures 6A, B**), suggesting that the prognostic model in this study could effectively predict OS and CSS in patients with OCCC.

## 4 Discussion

In the current study, according to stepwise Cox regression analyses, we screened out risk factors separately related to OS and CSS of OCCC patients. By comparing *AICc* scores, nomograms were constructed to assess the 1-, 3- and 5-year CSS and OS based on the identified prognostic factors (25). AUC, calibration curves and DCA curves in both training and validation sets showed favorable discrimination and calibration, indicating that our nomograms had good calibration power. Each risk factor included in the nomograms was attributed a risk score and was applied to successfully build a risk stratification system for predicting the OS and CSS of OCCC patients. Generally, younger age implied a better prognosis in EOC patients due to stronger immune response and better physical fitness (26, 27). However, our result indicated that OCCC patients younger than 45 years tended to have poorer prognosis. This result was in line with those of previous studies (28), which indicated the effect of age in OCCC may be different from other EOC. Moreover, we found a significant difference in the prognosis of OCCC patients in different marital statuses. Specifically, the prognosis of unmarried OCCC patients was worse compared to those who were married, which is the same as the finding of Kravdal et al (29). In this regard we generate the following analysis. Firstly, the companionship needs of married patients are met, and previous studies have shown that patients tend to be more emotionally positive when emotional needs are met. Therefore, MS may influence the prognosis of OCCC patients through emotions (30, 31). Secondly, Nayeri and colleagues found that married individuals tend to be diagnosed with cancer at an early stage (32).

The *AJCC* N-staging, a two-category system (N0: no regional lymph node metastasis; N1: histologically confirmed retroperitoneal lymph node metastasis), is the most basic and widely used cancer staging system and plays a vital role as a key prognostic factor in the development of postoperative treatment plans as well as in follow-up (33–35). However, this LN staging system does not account for the prognostic impact of PLNs and the number of RLNs. In fact, Nie et al. found that an increase in the number of PLNs is associated with lower DFS as well as OS (36). There is increasing evidence that the extent of LN dissection is also associated with the prognosis of patients with EOC (37). Therefore, the current LN staging appears inadequate in providing physicians with sufficient valuable information. Both LNR (the ratio of PLNs/RLNs) and LODDS take into account the number of PLNs and RLNs and both are more accurate than the pN staging system in predicting prognosis in several tumors (38, 39), but it is controversial which one is more superior (40, 41). There are many drawbacks of LNR led us to choose LODDS as the LN staging tool for this study. First, when the value of LNR is 0, its applicability is limited (e.g., 1/1 vs. 30/30). As the number of RLNs increases, the risk of post-op complications such as infection, vascular/nerve injuries, lymphatic leakage and lymphoedema increases, thus affecting patient prognosis (42). Then, the prognosis of patients may be significantly different despite having the same LNR (e.g., 1/2 vs. 15/30). Third, as mentioned, the majority of OCCC patients were still in stage I at the time of diagnosis (6). The probability of LN metastasis in early OCCC is relatively low, with only 3.6% in pT1aM0 and pT2aM0, compared with 71.6% in HGSOC (43). Compared with LNR, LODDS also has a unique value in the prognostic assessment of LN-negative patients (44). The value of LODDS increases with the decrease of RLNs. Additionally, there is an active debate about systematic lymphadenectomy in early-staging OCCC (45, 46). However, considering the calculation method of LODDS mentioned above, the clinician only needs to obtain the number of RLNs and the number of PLNs respectively to achieve the accurate value of LODDS. Therefore, LODDS acquisition does not depend on systematic lymphadenectomy. This will greatly reduce the difficulty of the surgery and the postoperative complication rate.

Several studies have found that the applications of nomogram models in several tumors have a better prognostic performance than the staging systems alone (47–49). With these nomograms, doctors can calculate the risk score for each patient, allowing for individualized prognostic assessment and guides postoperative personalized treatment. The AUC of the training and validation cohorts of the nomogram developed in our study was over 0.7, with the calibration points were separated on both sides of the ideal line. This means that we can obtain a more reasonable and more accurate follow-up schedule. Based on the results of the DCA curves, we believe that our model has higher discriminatory power than the traditional multivariate Cox regression.

It should be noted that there are several limitations in this study. First, while the SEER database certainly has a larger volume of data compared to prior case-series reports, it lacks records of some key variables related to prognoses, such as specific chemotherapy protocols, preoperative comorbidities, or postoperative complications. It is worthy to note that in this study we used part of the training set as the internal validation set, which does run the risk of producing an overly optimistic assessment of the efficacy of the predictive model. Although data from the real world supported our results, we will seek to re-evaluate the efficacy of our model in the future using completely independent data sets of larger sample sizes. Then, selection bias was inevitable due to the study’s retrospective nature. Fourth, statistical analyses were performed without correction for multiple testing, which may lead to potential false positives in the survival analysis.

## Data availability statement

The original contributions presented in the study are included in the article/**Supplementary Material**. Further inquiries can be directed to the corresponding author.

## Author contributions

ZL: Writing – original draft, Software, Methodology, Formal analysis, Conceptualization. CJ: Writing – review & editing, Validation, Methodology. YH: Writing – original draft, Methodology, Investigation. HY: Writing – original draft, Software, Visualization, Validation. ZC: Writing – review & editing, Visualization, Data curation. FK: Writing – review & editing, Supervision.
