# Supplementary material for: Construction and validation of log odds of positive lymph nodes (LODDS)-based nomograms for predicting overall survival and cancer-specific survival in ovarian clear cell carcinoma patients
Source: Front Oncol. 2024 Mar 21;14:1370272. doi: 10.3389/fonc.2024.1370272 (PMC10991783; doi:10.3389/fonc.2024.1370272)
Supplement: Supplementary file 1 [file DataSheet_1.pdf]

Supplementary Material

1 Supplementary Figures and Tables

1.1 Supplementary Figures

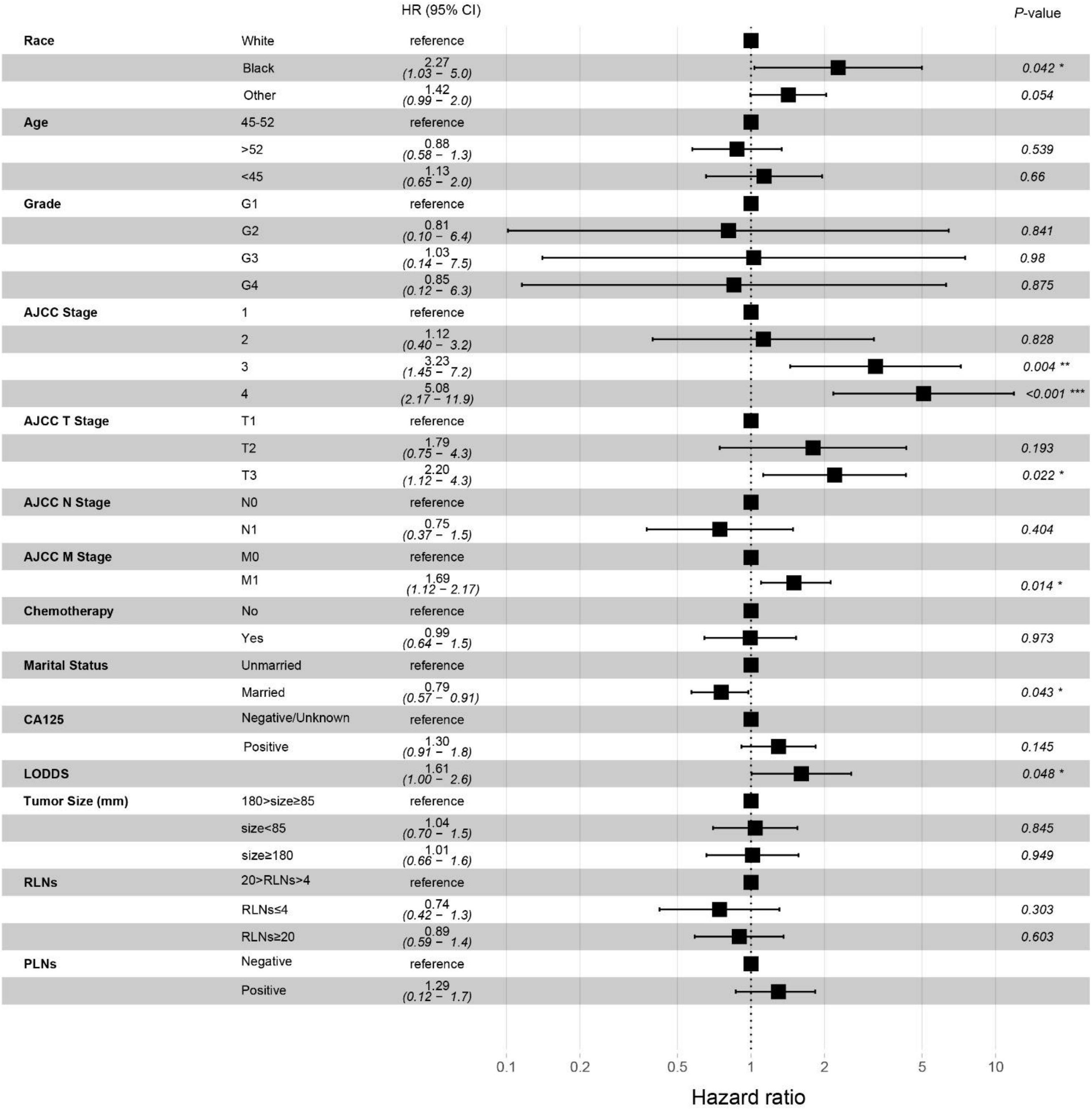

Supplementary Figure 1. Forest plots of independent risk factors in multivariate Cox regression analysis of OS.

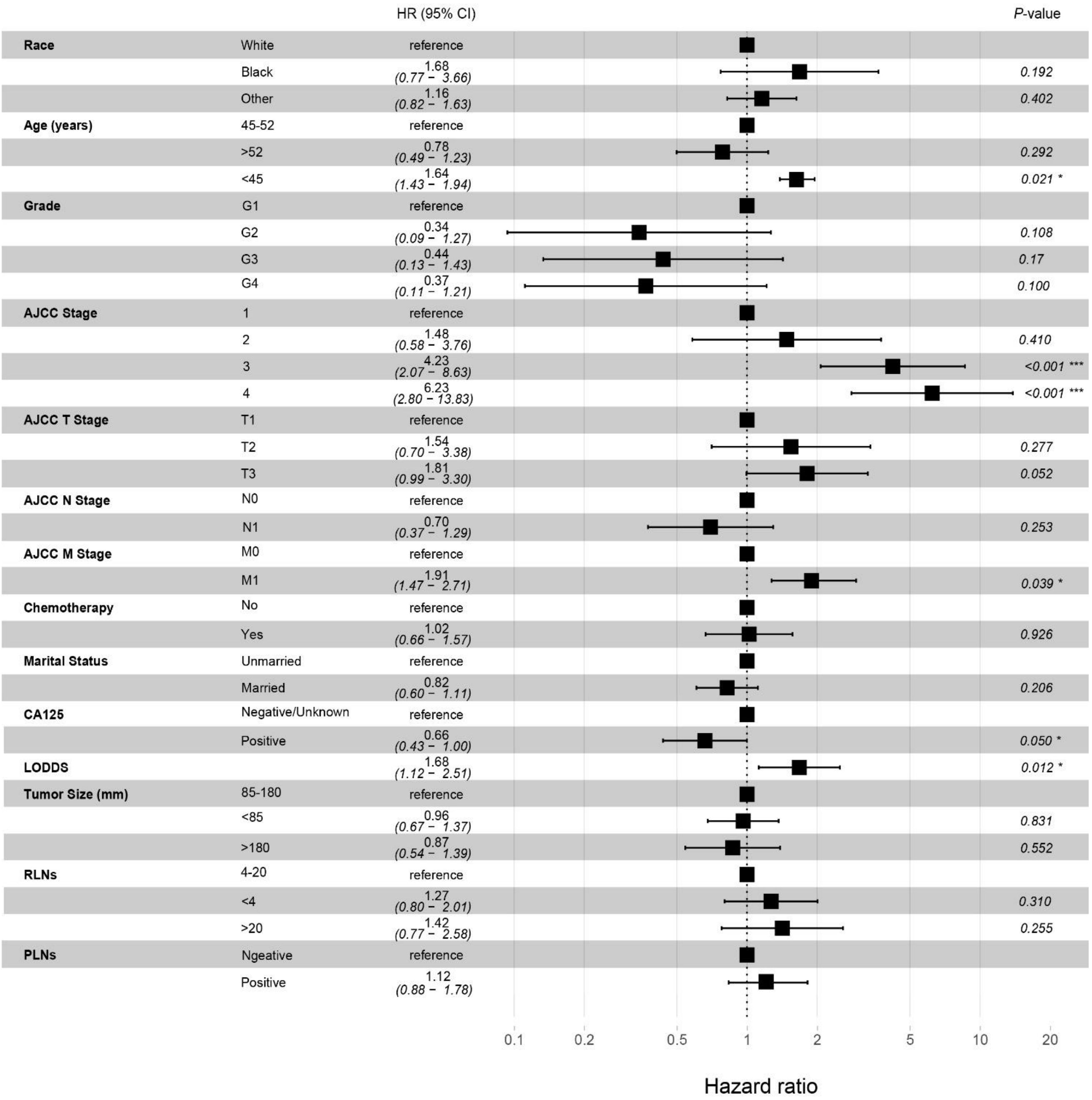
HR (95% CI)
P-value
Race
White
reference
Black
1.68 (0.77 – 3.66)
0.192
Other
1.16 (0.82 – 1.63)
0.402
Age (years)
45-52
reference
>52
0.78 (0.49 – 1.23)
0.292
<45
1.64 (1.43 – 1.94)
0.021 *
Grade
G1
reference
G2
0.34 (0.09 – 1.27)
0.108
G3
0.44 (0.13 – 1.43)
0.17
G4
0.37 (0.11 – 1.21)
0.100
AJCC Stage
1
reference
2
1.48 (0.58 – 3.76)
0.410
3
4.23 (2.07 – 8.63)
<0.001 ***
4
6.23 (2.80 – 13.83)
<0.001 ***
AJCC T Stage
T1
reference
T2
1.54 (0.70 – 3.38)
0.277
T3
1.81 (0.99 – 3.30)
0.052
AJCC N Stage
N0
reference
N1
0.70 (0.37 – 1.29)
0.253
AJCC M Stage
M0
reference
M1
1.91 (1.47 – 2.71)
0.039 *
Chemotherapy
No
reference
Yes
1.02 (0.66 – 1.57)
0.926
Marital Status
Unmarried
reference
Married
0.82 (0.60 – 1.11)
0.206
CA125
Negative/Unknown
reference
Positive
0.66 (0.43 – 1.00)
0.050 *
LODDS
1.68 (1.12 – 2.51)
0.012 *
Tumor Size (mm)
85-180
reference
<85
0.96 (0.67 – 1.37)
0.831
>180
0.87 (0.54 – 1.39)
0.552
RLNs
4-20
reference
<4
1.27 (0.80 – 2.01)
0.310
>20
1.42 (0.77 – 2.58)
0.255
PLNs
Ngeative
reference
Positive
1.12 (0.88 – 1.78)
0.1 0.2 0.5 1 2 5 10 20
Hazard ratio

Supplementary Figure 2. Forest plots of independent risk factors in multivariate Cox regression analysis of CSS.

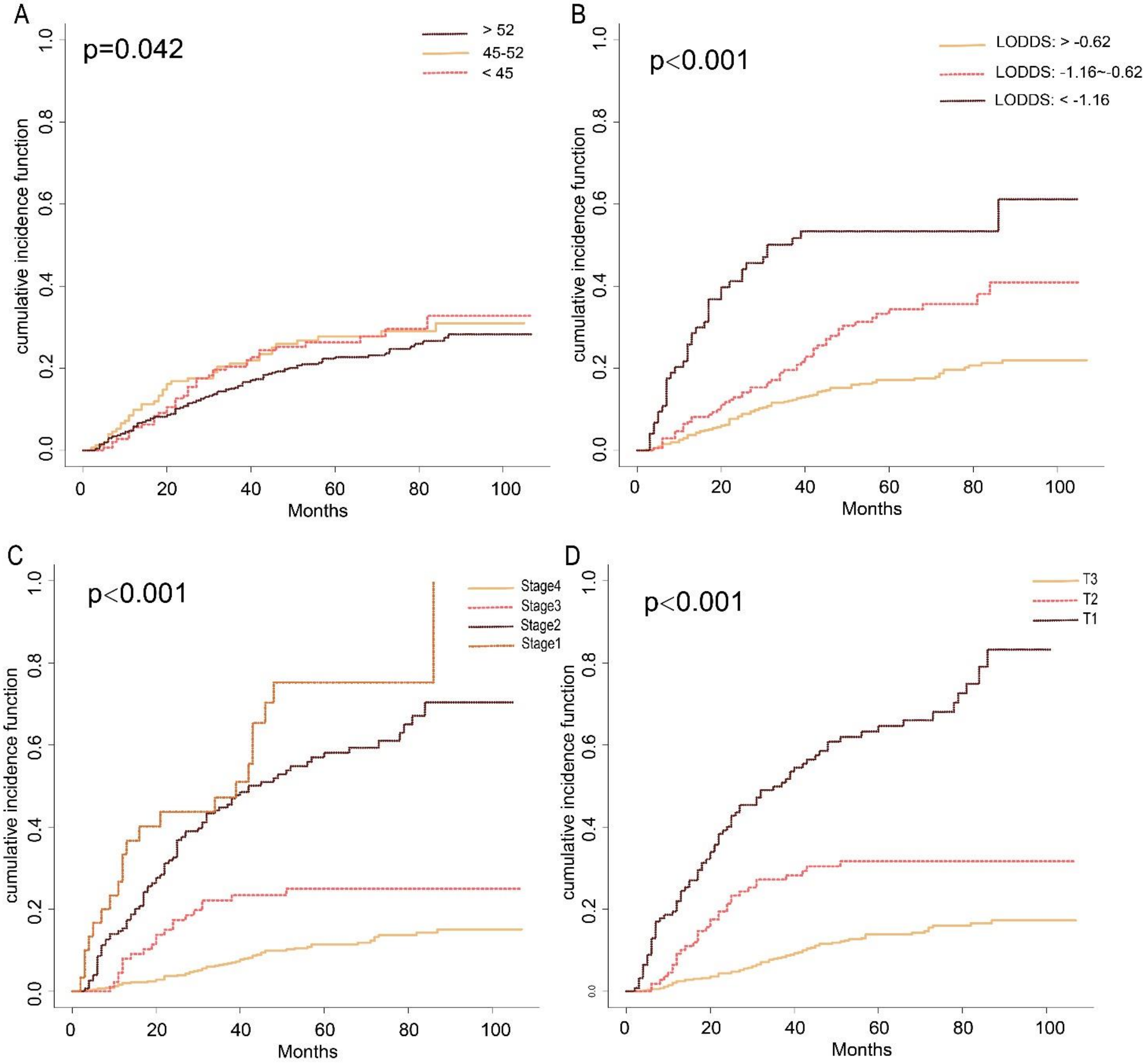

Supplementary Figure 3. Cumulative incidence curves for CSS, stratified by age (A); LODDS (B); stage (C); 7th AJCC T Stage (D).

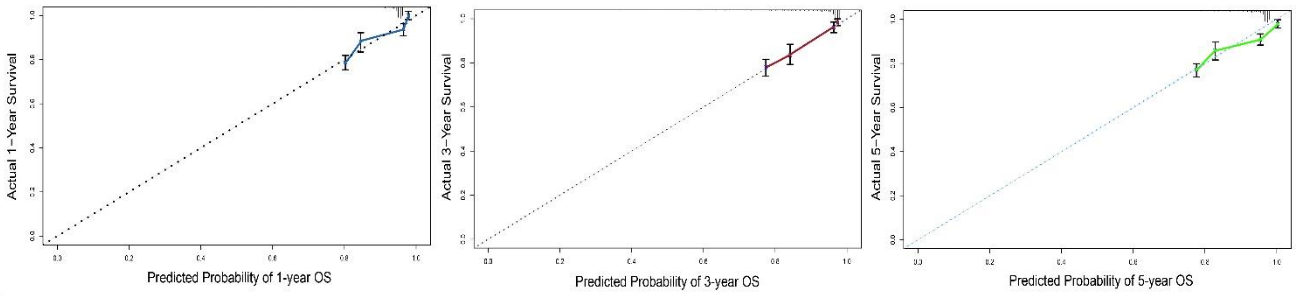

Supplementary Figure 4. 1-, 3- and 5-year OS nomogram calibration plots for training cohort.

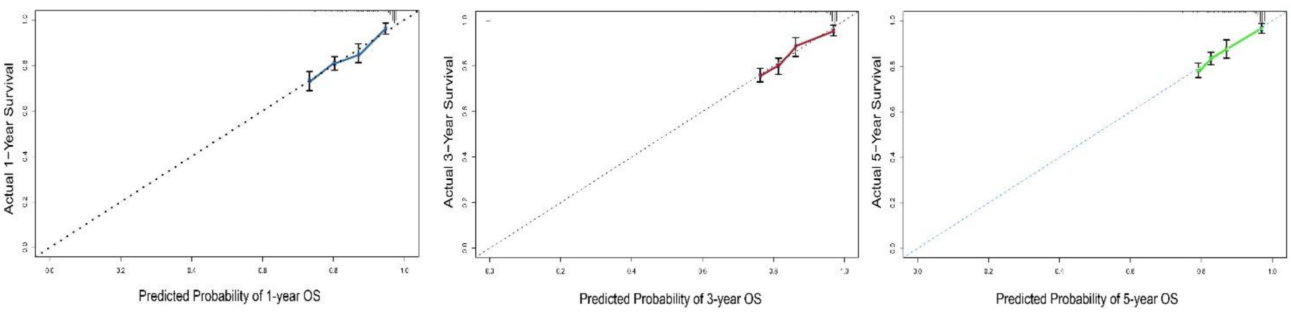

Supplementary Figure 5. 1-, 3- and 5-year OS nomogram calibration plots for internal validation cohort.

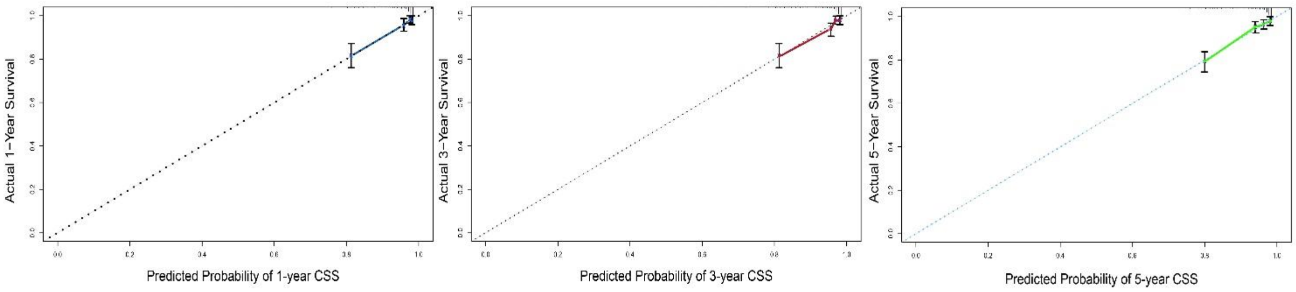

Supplementary Figure 6. 1-, 3- and 5-year CSS nomogram calibration plots for training cohort.

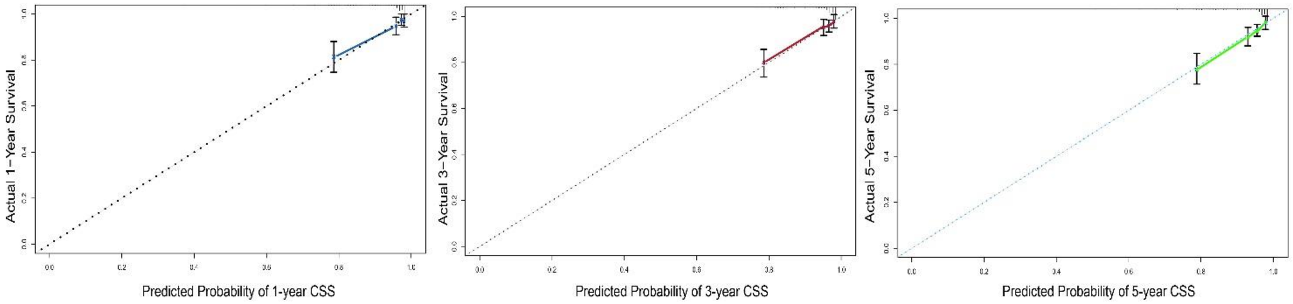

Supplementary Figure 7. 1-, 3- and 5-year CSS nomogram calibration plots for internal validation cohort.

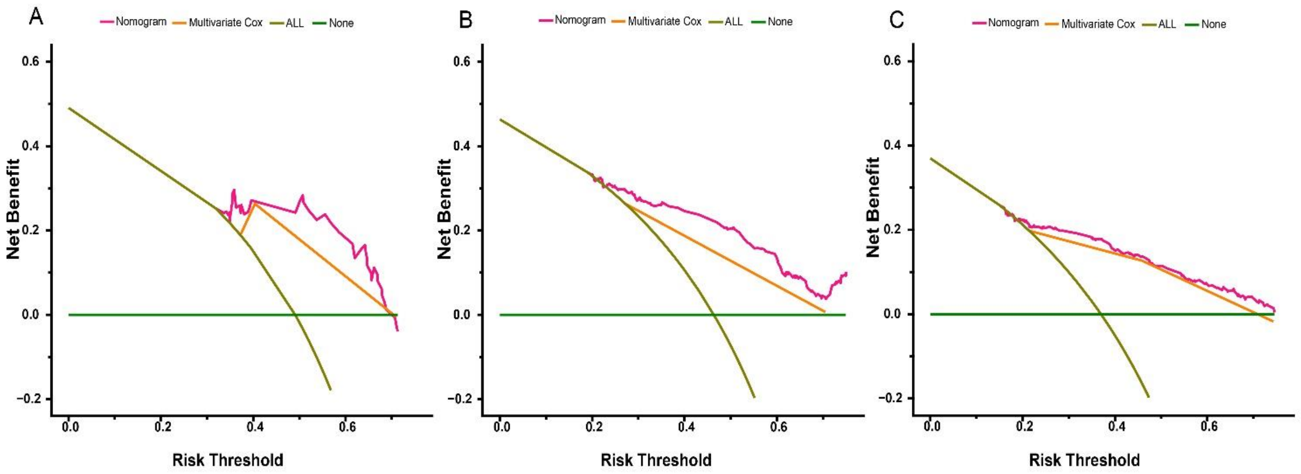

Supplementary Figure 8. DCA curve of the nomogram and multivariate *Cox* analysis. 1-year (A), 3-year (B) and 5-year (C) OS.

DCA: decision curve analysis.

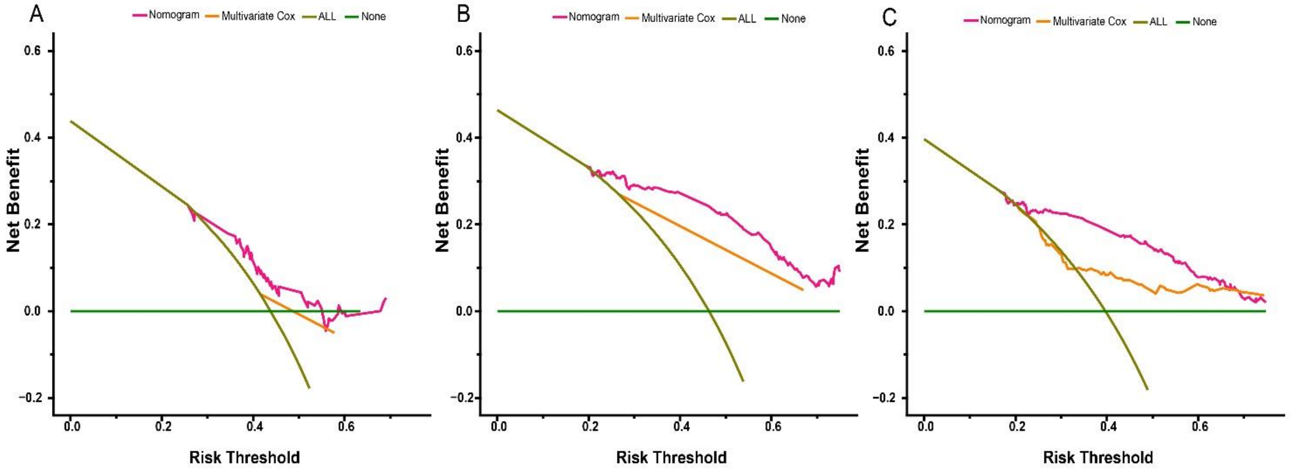

Supplementary Figure 9. DCA curve of the nomogram and multivariate *Cox* analysis. 1-year (A), 3-year (B) and 5-year (C) CSS.

DCA: decision curve analysis.
